# An Unique Case of Cancer

**Published:** 1871-08

**Authors:** Norman Bridge

**Affiliations:** Chicago


### AN UNIQUE CASE OF CANCER.

By N. BRIDGE, M.D., Chicago.

[Read before the Chicago Medical Society, July 31, 1871.]

The following case is submitted as being unusual, and hence instructive, as perhaps throwing some light on the possible influencing of cancers by medicines.

Mrs. Alice A. S., aged about 65 years, short and slender, had been through life usually healthy; had borne nine children, and was always active and given to work.

In June, 1864, after it had been a year in developing, she had a scirrhus cancer, as diagnosed by Dr. D. Brainard, removed from the left breast, at the hands of that surgeon. From this she soon recovered and enjoyed tolerable health, until about two and a-half years ago—except that she had cataract in both eyes, beginning five or six years ago,—when she began to notice a reappearance of the disease in the left breast, or that part of it unremoved; soon after, a tumor was noticed in the right breast. The growths did not develop rapidly, and she submitted herself to treatment by a "cancer doctor," in June, 1869; this man soon died, and she went to New Jersey to another charlatan, whose treatment was similar to the first, remaining under his care until she lost all hope of recovery, when, in December, 1869, she returned to Chicago. Although not then a great sufferer, she had been for some months getting "short of breath," especially on any exercise. This steadily grew worse until by the 15th of March, 1870, when I was first called, she found considerable difficulty in breathing and breathed very rapidly; she had pain in the precordium, and some pain and uneasiness in the stomach, and annoying eructions of gas after eating. The appetite was impaired; the bowels moved regularly; she had pains, some discribed as "shooting" through the chest. The cancerous cachexia was well marked.

On auscultation and percussion the right lung appeared to be, through its lower half, mostly solidified; there was almost complete dulness over this space, and coarse ronchi and bronchial breathing only could be heard. There was evident effusion into the plural cavity, but its quantity was small, and it reached not more than to the middle third of the lung, as the body was erect. In the upper half, there was coarse rales and ronchi, and exaggerated lung sound.

Both resonance and respiratory murmur were increased over the left side. The pulse was about 85, and respiration 25, when at perfect rest. At this time the tumor in the right breast was large, the nipple was retracted, axillary glands swollen, and all the signs of rapidly growing scirrhus were evident in both this and the remaining portion of the left breast.

The patient was ordered, for its effect on the stomach and to test its possible effect on the cancer, one grain of carbolic acid daily, in divided doses. A tonic was given of elixir of calisaya and iron, with quinine added, so that two or three grains of the latter would be taken daily. An anodyne of opium was ordered for each night.

The stomach symptoms soon abated, the breathing became easier, and in a few days the appetite was much improved. But in two weeks the strength began to fail, the breathing to be more rapid and difficult. The patient continued to sink until the beginning of May, when I left town for a vacation. At this time she was obliged to keep her bed most of the time; the appetite was entirely lost. Physical exploration demonstrated the rapid increase of the solidification of the right lung. Dr. J. P. Ross, whom I asked to see the case with me, concurred in this conclusion.

Once, about a month after treatment was begun, severe neuralgic pains in the vulva, mouth of urethra, and in adjacent parts, were set up, which were erratic; they would attack her in an instant, like an electric shock, and as quickly depart. Carbolic acid, morphine, and olive oil, applied externally, gave relief.

Up to the time of my departure, no change was made in the general medication.

On the last of April, the respiration was 32, and the pulse 100.

On returning, June 6th, patient was found better; pulse 96, respiration 30; a little appetite. The local neuralgia was worse; use was made of the former mixture with little effect. Various local anodynes were employed, and finally, with best effect, an ointment of equal parts extracts of aconite, belladonna, cannabis indica, and hyoscyamus. The opium given internally was increased.

The right side of the chest was almost entirely solid to auscullation and percussion; on that side there was no motion of the chest in respiration. The tumors in the breasts had ceased to grow. The tonic and carbolic acid treatment was from this time kept up with little change, save a decrease in the carbolic acid, the patient remaining about the same and failing hardly at all, until the 3rd of March, 1871, when she began to fall away rapidly. Breathing became oppressive, pain and restlessness increased. The opiate was increased, and in a few days she was better. The tonic medicine was stopped, and pills of strychnia of 1-10 grain each were given, one each day, with, in two weeks, very good effect.

On April 16th, the œdema, which had existed in the lower limbs for a number of weeks, was noticed to be worse, and worse in the left than the right limb. The pulse was 85, and respiration 25 per minute. The tumor in the right breast had decreased to not more than one-sixth its former size, and was flat and hard, and the nipple had shriveled up and dropped off, leaving no ulcer. The tumor in the left side had proportionately decreased. Pain in both breasts had been absent many months.

For three weeks before death the patient had been troubled with nausea, and had complained that her food only went down as far as the middle of the sternum.

She steadily grew weak, and died on the 16th of June.

A *post mortem* examination was made twenty hours after death. The pleura lining the right lung cavity was found very much thickened, the mediastinal and diaphragmatic portions being much more thickened than that attached to the ribs; the former, in places, was over a quarter of an inch thick, and was as dense and resisting as thick leather; the increase in the structure beneath the lung was partly of the diaphragm, this structure being hypertrophied as far as the œsophagus, and constricting somewhat the calibre of that tube.

The plural cavity was filled with a brownish limpid fluid; the lung was contracted to the size of a child's fist, and was drawn up near the clavicle; its pleural covering was somewhat thickened, light colored, and indurated. The lung itself was dense and black; it contained a few cancerous masses, but most of its substance was solid, simply from the pressure evidently made upon it. The larger bronchial tubes only extended, patulous, into the lung, and these but for a short distance; they were through the rest of their extent, collapsed by the outside pressure. There was no bulging of the right pleural sac, in any direction, and, on emptying it, there was no disposition to collapse.

The left lung was pathological only in having a large pigmentary deposit; it was of the normal size and had no deposits of cancer or tubercle.

The liver was light colored, mottled and fatty, but not enlarged. The pancreas was enlarged, abnormally lobulated and fatty.

The mucous membrane of the stomach was slightly softened, but not cancerous; neither was the œsophagus.

On microscopical examination—in which the skill and experience of Dr. I. N. Danforth were invoked to aid me—the thickened pleura was found to be cancerous, made up of dense fibres and thickets of scirrhus cells. Abundant cancer cells were found in the liver, but none in the pancreas.

The shriveled tumor in the right breast gives the microscopic characteristics of cancer.

This case seems to me peculiar in several particulars.

It exhibits a scirrhus tumor of the breast, which, after growing rapidly for months, and giving all the signs, in size, hardness, retraction of nipple, and the like, to indicate a probable early breaking down in ulceration, begins to decrease in size soon after treatment is commenced, and in fifteen months has shriveled to an insignificant mass. In the tumor too, the pains entirely disappear when the size begins to decrease.

We see here too, the secondary deposit of cancer mainly in the pleura, and tissues beneath it, the cavity of which is filled with fluid, insomuch, that, with the aid of a slight contraction and thickening of the pleura pulmonalis, the lung is squeezed —so far as concerns its functions—literally out of existence; so that, in opening the cavity, the doctors exclaim that the lung has disappeared, and only after some hunting it is found; on section, its appearance is as though a lung full of black pigment were squeezed in a vice.

Moreover, the patient, six weeks after beginning treatment, ceases to lose strength, and is kept, for a year, from failing.

This curious behavior of the case occurs when carbolic acid is being regularly taken; the present discussion of the effect of this agent on cancer makes this an important consideration in the case.

This patient took, during the last fifteen months of her life, not far from 300 grs. of carbolic acid. Did it have any effect on the disease, or would this have run the course it did without it?

There are but few cases on record where a scirrhus breast, after attaining any size, ever became shriveled and indurated spontaneously. Errichsen refers to the fact, but in such a way, as to leave us in doubt whether he means a case of this sort, or one where the tumor is always small and hard, and not a large mass grown small.

In Holmes, the spontaneous reduction in size, of a cirrhus tumor, is recorded as a fact, albeit of very rare occurrence.

In view of the accumulating testimony to the efficacy, or the "seeming" efficacy of carbolic acid in cancer, if in this case it did not influence the course of the disease, then it is a remarkable concidence.

While there is no sufficient ground for asserting positively that it did effect the cancer, the case certainly helps to warrant us in testing further its power in this disease.

